# MiR-361-5p inhibits glycolytic metabolism, proliferation and invasion of breast cancer by targeting FGFR1 and MMP-1

**DOI:** 10.1186/s13046-017-0630-1

**Published:** 2017-11-13

**Authors:** Fei Ma, Lei Zhang, Li Ma, Yiyun Zhang, Jianguo Zhang, Baoliang Guo

**Affiliations:** 1 Department of General Surgery, the Second Affiliated Hospital of Harbin Medical University, 246 Xuefu Street, Nangang District, Harbin, China; 2 Department of Ultrasound, the Second Affiliated Hospital of Harbin Medical University, Harbin, China; 3 Computer Center, the Fifth Hospital of Harbin, Harbin, China; 4 Department of Endoscopy, Harbin Medical University Cancer Hospital, Harbin, China

**Keywords:** miR-361-5p, Glycolysis, FGFR1, MMP-1

## Abstract

**Background:**

MicroRNAs function as key regulators in various human cancers, including breast cancer (BC). MiR-361-5p has been proved to be a tumor suppressor in colorectal cancer and gastric cancer in our previous study. In this study, we aim to find out the function of miR-361-5p in breast cancer progression and elaborate the mechanism that miR-361-5p acts its function in breast cancer.

**Methods and results:**

Here we reported that miR-361-5p was down-regulated in breast cancer tissue compared with normal breast tissue and the expression of miR-361-5p was positively associated with prognosis in BC patients. Functional studies showed that overexpression of miR-361-5p suppressed the proliferation, invasion and metastasis of breast cancer cells both in vivo and in vitro. Mechanistically, we found that miR-361-5p inhibited the proliferation of BC cells by suppressing glycolysis. FGFR1, a promoter of glycolysis-related enzyme, was identified as the target of miR-361-5p that promoted glycolysis and repressed oxidative phosphorylation. Furthermore, we demonstrated that miR-361-5p inhibited breast cancer cells invasion and metastasis by targeting MMP-1. An inverse expression pattern was also found between miR-361-5p and FGFR1 or MMP-1 in a cohort of 60 BC tissues.

**Conclusion:**

Our results indicate that miR-361-5p inhibits breast cancer cells glycolysis and invasion by respectively repressing FGFR1 and MMP-1, suggesting that miR-361-5p and its targets may serve as therapeutic targets in breast cancer treatment.

## Background

Warburg effect was first described as a common metabolic feature of cancer cells almost 90 years ago, which has also been known as aerobic glycolysis nowadays [1]. This phenomenon indicates that cancer cells tend to consume more glucose to produce lactate by glycolysis rather than oxidative phosphorylation even in oxygen-rich conditions [2]. This metabolic shift is thought to provide diverse glycolytic intermediates for anabolic biosynthesis instead of energy production in rapidly proliferating cancer cells [3]. Thus, the understanding of controlling the shift from oxidative phosphorylation to aerobic glycolysis is crucial for cancer treatment.

At present, breast cancer (BC) is the most prevalent cancer among women in China and the incidence of BC is still increasing rapidly [4]. Despite numerous evidence have shown that accumulation of genetic and epigenetic changes cause tumorigenesis and progression [5], the mechanisms underlying the pathogenesis of BC remain to be clearly defined. Given that recrudescence and metastasis occur frequently and associate closely with BC death [6], understanding the fundamental mechanism that facilites cancer progression and finding new sights in breast cancer treatment are of great importance.

MicroRNAs (miRNAs) are a class of small non-coding RNAs that can play central regulatory roles in the development of breast cancer [7]. They can imperfectly pair with the 3′-untranslated region (UTR) of their target mRNAs and trigger mRNAs degradation or translation inhibition [8]. It has been evidenced that miRNA expression is closely associated with tumor proliferation and metastasis [9]. For example, miR-146a and miR-301a promotes breast cancer progression by targeting EMT markers and PTEN, respectively [7, 10]. Positive expression of miR-361-5p has been proved to indicate better prognosis for BC patients [11]. However, the specific function and regulatory mechanism of miR-361-5p in BC progression is rarely investigated. In this study, we sought to reveal how miR-361-5p exerts influence on BC progression, identify and characterize its target genes.

## Methods

### Cell lines and cell culture

Human spontaneously immortal cell line and breast cancer cell lines, including MCF-10A, MCF-7, MDA-MB-231, MDA-MB-468, T47D, MDA-MB-549 and HEK-293 T were cultured under conditions recommended by ATCC. The cells were maintained in DMEM (Hyclone) supplemented with 10% FBS (Hyclone) at 37 °C under an air atmosphere containing 5% carbon dioxide.

### RNA extraction and RT-PCR

Total RNA were extracted and reverse transcribed by using TRIZOL reagent (Invitrogen) and M-MLV RT kit (Promega). For detecting miR-361-5p, the Mir-VanaTM MiRNA Isolation Kit (Ambion, USA) was used to isolate total RNA from cell lines and patient samples following the manufacturer’s instructions. MiR-361-5p was detected using Platinum Taq DNA Polymerase (Invitrogen) with specific primers: miR-361-5p forward: ATAAAGRGCRGACAGTGCAGATAGTG, miR-361-5p reverse: TCAAGTACCCACAGTGCGGT, and U6 forward: CTCGCTTCGGCAGCACA, U6 reverse: AACGCTTCACGAATTTGCGT. Results were expressed as fold change using the 2-**△△**CT method.

### Plasmid construction and transfection

For the stable transfection of anti-miR-361-5p, anti-miR-361-5p sequence were amplified from miRZip-361-5p construct (System Biosciences) and cloned into pSilencer4.1 system. BC cells were then transfected with the pSliencer vector containing the antisense sequence of miR-361-5p. The cells were selected by puromycin after 48 h transfection and then diluted. MiR-361-5p mimics, miR-control, FGFR1 siRNA, MMP-1 siRNA or siRNA negative control were purchased from Genepharma (China). FGFR1 and MMP-1 cDNA ORF Clone were purchased from Origene (Origene Technology). Transient transfections were performed by using Lipofectamine 2000 (Invitrogen) following the manufacturer’s protocol. Cells were kept in medium containing 2% FBS for 48 h and then harvested and used.

### Luciferase reporter assay

HEK293T cells were used to perform the luciferase reporter assay. FGFR1 3′-UTR, mutated FGFR1 3′-UTR, MMP-1 3′-UTR, mutated MMP-1 3′-UTR, or control luciferase reporter plasmid was cotransfected with miR-361-5p mimics or negative control (Ambion) using Lipofectamine 3000 (Thermofisher, USA). Luciferase activity was measured by SecrePair Dual-Luciferase Reporter System (GeneCopoeia).

### Western blot

Indicated cells were lysed with RIPA buffer. Protein lysates were electrophoresed through SDS polyacrylamide gel, followed by transferring to PVDF membranes (Millipore, USA). The membranes were blocked with non-fat dry milk at room temperature and then incubated with primary antibodies at 4°Covernight. The primary antibodies included: FGFR1, PDHK1, P-PDHK1, LDHA, P-LDHA, MMP-1 and β-actin (Cell Signaling Technology). Membranes were then washed and incubated with secondary antibodies. Proteins were visualized by the electrochemiluminescence (ECL) western blot substrate detection (Pierce).

### Cell proliferation and colony formation assays

The xCELLigence RTCA DP (Roche) instrument was used to perform the real-time cell proliferation assays according to the manufacturer’s instructions. The concomitant changes in Cell Index reflected the changes in cell numbers. For colony formation assays, 1 × 10^3^ cells were seeded in a well of a 6-well plate and cultured for 1–2 weeks. The cells were then fixed and stained. The cell colonies were imaged and analyzed.

### Cell invasion assay

For transwell invasion assays, 2 × 10^5^ cells were plated into the upper chamber of the insert (Corning) coated with Matrigel (BD Bioscience). Cells were seeded in medium without serum in the upper chamber and the medium in lower chamber was supplemented with 10% FBS. Cells were cultured for 48 h and cells invaded to the underside of the membrane were fixed, stained, imaged and counted.

### Cellular glucose-6-phosphate assay

The levels of glucose-6-phosphate in indicated cells were measured using Glucose-6-phosphate Fluorometric Assay kit (Cayman, USA). All results were normalized to total protein expression levels.

### Measurement of the extracellular acidification rate (ECAR)

The extracellular acidification rate (ECAR) was detected using a Seahorse Bioscience XF24 extracellular flux analyzer (Seahorse Bioscience). The cartridge sensor was hydrated overnight with buffer at 37 °C without CO2. Indicated cells were plated in an XF24 Islet Capture Microplate and the medium was replaced with serum-free DMEM/F12 without sodium bicarbonate. ECAR values were observed under basal conditions and measured after the input of oligomycin (1 μM), FCCP (1 μM), and antimycin A (1 μM) into the well. ECAR values were analyzed by using the Seahorse XF-24 software. Every point represents an average of five different wells.

### Glucose consumption and lactate production

Glucose consumption and lactate production were measured by using the supernatant of the indicated cells. The glucose assay kit (Sigma-Aldrich, St. Louis, MO, USA) was used to measure glucose levels following the manufacturer’s instructions. The lactate production was determined by the colorimetric lactate kit (Bio Vision, Milpitas, CA, USA). The concentrations of metabolite were examined on deproteinized samples by performing specific enzymatic assays by a CMA600 analyzer (CMA Microdialysis AB, Sweden). The results were normalized to protein content using the Pierce BCA Protein assay (Thermo Scientific).

### Immunofluorescence assay

Indicated cells were fixed with formaldehyde and permeabilized with 0.2% Triton X-100. After blocked with 10% goat serum, the indicated cells were incubated with primary antibodies and then corresponding secondary antibodies (Cell Signaling Technology). Images were taken using Zeiss confocal microscopy.

### Animal studies

Balb/c nude mice and SCID mice were purchased from Vital Rivers (Beijing, China) and maintained under SPF conditions. All experiments involving animals were performed in accordance with the Guide for the Administration of Affairs Concerning Experimental Animals, the national guideline for animal experiments. Cells were suspended in culture medium. A 160 μl sample of medium containing 1 × 10^7^ cells was injected into the dorsal flank of nude mice subcutaneously. The growth of tumor was monitored every week and the tumor xenografts were collected and weighed after 5 weeks. For lung metastasis model, 2 × 10^7^ cells were injected through the tail vein of SCID mice. After 5 weeks, the mice were injected with luciferin through tail vein 10 min before imaging. Imaging was performed by the Xenogen IVIS Spectrum Imaging System (Caliper Life Sciences, USA). Then the mice were sacrificed and the lungs were collected for detection. The number of metastatic nodules and tumor volume were evaluated. For each tissue, HE staining was performed for histological examination. All the animal experiments were approved by the Animal Experimental Ethics Committee of Harbin Medical University.

### Clinical samples

Sixty pairs of primary breast cancer and corresponding normal breast tissues were collected and conserved in −80 °C condition after breast resection and pathological confirmation between November 2005 and March 2006 in the Second Affiliated Hospital of Harbin Medical University. The patients should not receive chemotherapy or radiation therapy before BC resection in this study. This study was performed according to the ethical standards of Declaration of Helsinki and all the patients provided written informed consent for the use of tissues. The TNM stage was determined in accordance to the classification proposed by the AJCC Cancer Staging Manual.

### In situ hybridization and immunohistochemistry

In situ hybridization and immunohistochemistry staining were performed as previously described [12].

### Statistical analysis

Results were presented as mean ± standard deviation from at least three replicates. The Student’s t-test was used to compare differences between groups. Statistical analysis was performed by GraphPad Prism 5 software. Significant data were indicated by asterisks *P* < 0.05 (*), *P* < 0.01 (**).

## Results

### The expression of miR-361-5p is decreased in breast cancer which is associated with poor prognosis

**Fig. 1:**
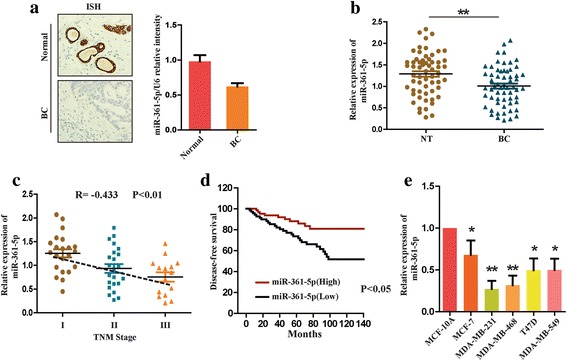
Downregulation of miR-361-5p in breast cancer and its association with poor prognosis. **a** ISH was used to compare the expression of miR-361-5p in BC tissues and corresponding normal tissues in a group of 60 patients. **b** Quantitative RT-PCR was used to detect the expression of miR-361-5p in BC tissues and corresponding normal tissues. **c** Relative expression of miR-361-5p was measured in BC tissues of different tumor stages by qRT-PCR. **d** Kaplan-Meier survival test was used to test the relationship between miR-361-5p expression and disease-free survival of BC patients. **e** The relative expression of miR-361-5p was detected in different BC cell lines by qRT-PCR. *P < 0.05, **P < 0.01

To decide the role of miR-361-5p in breast cancer, we first examined the expression levels of miR-361-5p in normal breast and BC tissues in a group of 60 BC patients by qRT-PCR and ISH. We found that miR-361-5p expression was significantly decreased in 49 of 60 BC tissues compared with corresponding normal breast tissues (Fig. 1a and b). To explore the clinical significance of miR-361-5p down-regulation in BC, we further detected the relationship between clinicopathologic data and miR-361-5p expression levels. It was observed that decreased miR-361-5p expression was correlated with larger tumor size and lymph node metastasis (Table 1). As shown in Fig. 1c, we also found that miR-361-5p expression was closely associated with TNM stage. Importantly, Kaplan-Meier analysis indicated that patients with higher miR-361-5p expression showed significantly better disease-free survival (Fig. 1d). We next examined the expression of miR-361-5p in different BC cell lines. The expression levels of miR-361-5p were found to be markedly lower in BC cells lines, especially highly metastatic cells (MDA-MB-231, MDA-MB-468), compared with spontaneously immortal MCF-10A cells (Fig. 1e). Altogether, these data suggest that the expression of miR-361-5p is decreased in breast cancer and its downregulation is associated with poor prognosis.

**Table 1:** Relationship between miR-361-5p expression and clinicopathologic features of BC patients (*n* = 60)

variable	Relative miR-361-5p expression		*P*-value
	Low (*n* = 30)	High (n = 30)	
Age			NS
< 50	17	14	
> 50	13	16	
Histological differentiation			NS
Well	7	6	
Moderate	14	17	
Poor	9	7	
ER status			NS
Positive	16	17	
Negative	14	13	
PR status			NS
Positive	21	14	
Negative	9	16	
Her-2 status			NS
Positive	11	8	
Negative	19	22	
Tumor size			*P* < 0.05
< 2 cm	11	21	
2-5 cm	12	6	
> 5 cm	7	3	
Lymph node metastasis			*P* < 0.01
Yes	21	6	
No	9	24	
Tumor stage			*P* < 0.05
I	7	15	
II	12	11	
III	11	4	
Molecular subtype			NS
Luminal like	16	17	
HER-2 positive	10	7	
Triple negative	4	6	

Note: BC patients were divided into miR-361-5p High group and Low group according to the analysis of qRT-PCR detection. NS, not significant between different groups. Differences among variables were evaluated by χ^2^ or Fisher’s exact χ^2^ -test

### MiR-361-5p suppresses breast cancer cells proliferation, invasion and metastasis both in vitro and in vivo

**Fig. 2:**
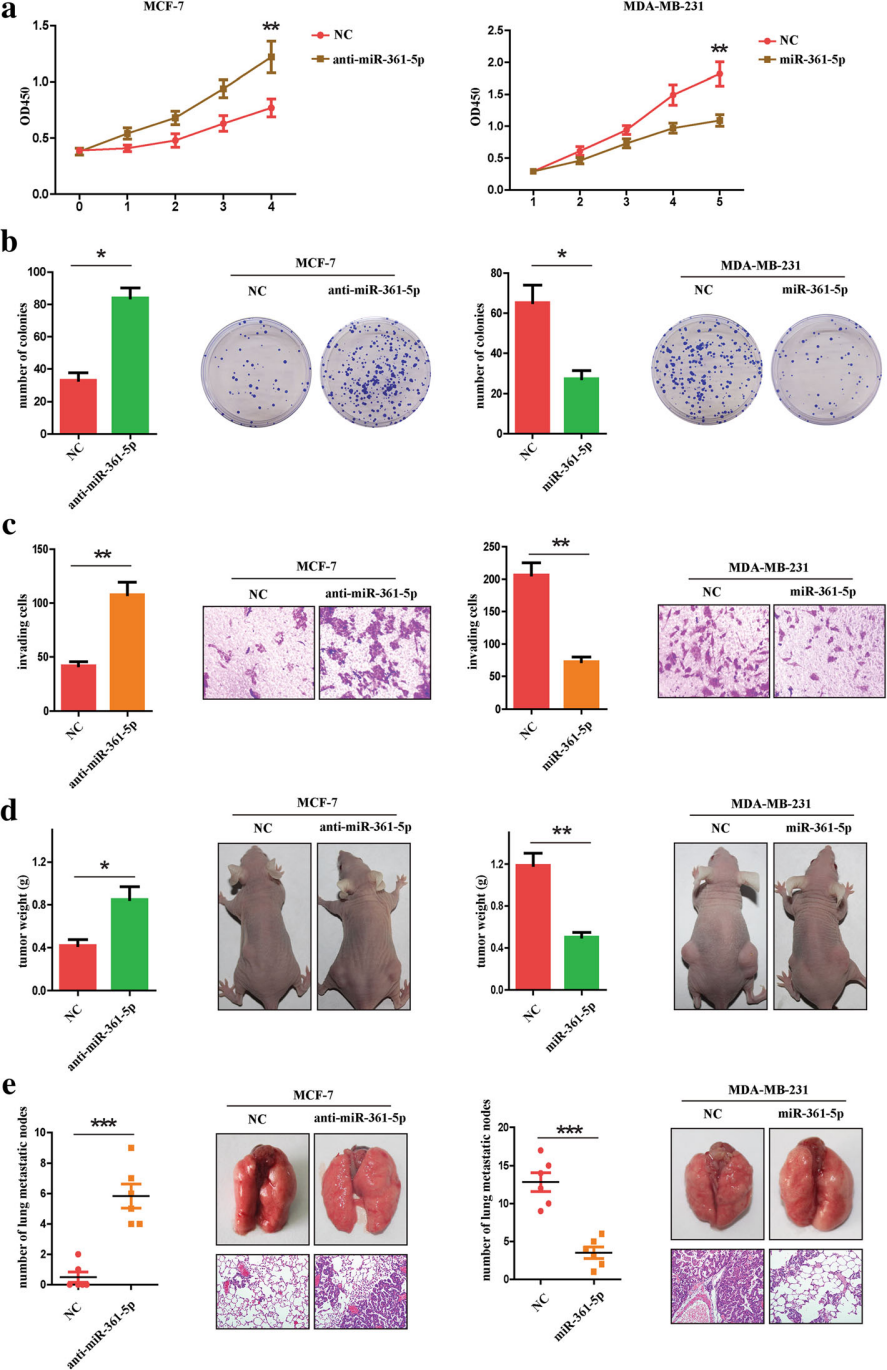
MiR-361-5p suppresses breast cancer cells proliferation, invasion and metastasis in vitro and in vivo. **a** Real-time cell proliferation assays in BC cells with miR-361-5p overexpression or inhibition. **b** Colony formation assays in BC cell with miR-361-5p overexpression or inhibition. **c** Effect of miR-361-5p on the invasion ability of BC cells using transwell assays. **d** The tumor weight in nude mice injected with indicated cells (left panel). Representative images of the mice injected with indicated cells (right panel). **e** Effect of miR-361-5p on tumor metastasis in SCID mice injected with indicated cells. The number of lung metastatic nodes in mice injected with indicated cells (left panel). Representative images of lung metastasis (right upper panel). Representative images of HE staining of the lung sections (right lower panel). *P < 0.05, **P < 0.01, ***P < 0.001

Given the correlation of miR-361-5p expression and prognosis in BC patients, we further detected the biology effect of miR-361-5p deregulation on breast cancer cells. As shown in Fig. 2a, real-time cell proliferation assays indicated that miR-361-5p overexpression significantly inhibited growth of MDA-MB-231 cells. Conversely, miR-361-5p inhibitors promoted the growth of MCF-7 cells. Colony formation assays showed similar results (Fig. 2b). Matrigel-coated transwell assays indicated that MCF-7 cells transfected with anti-miR-361-5p showed significantly enhanced invasion ability, whereas overexpressing miR-361-5p in MDA-MB-231 cells decreased the invasion ability of cancer cells (Fig. 2c). Consistent with the results in vitro, MCF-7 cells transfected with anti-miR-361-5p showed enhanced tumorigenic ability in vivo. In comparison with the control group, tumorigenic ability dramatically decreased in the miR-361-5p group (Fig. 2d). Meanwhile, we observed that mice injected with MDA-MB-231 cells overexpressing miR-361-5p exhibited less lung metastatic nodes compared with the control group. Conversely, transfection with anti-miR-361-5p in MCF-7 cells markedly increased the number of lung metastatic nodes in node mice. To conclude, miR-361-5p suppresses breast cancer cells proliferation, invasion and metastasis both in vitro and in vivo.

### MiR-361-5p inhibits glycolysis and increases mitochondrial metabolism in BC cells

**Fig. 3:**
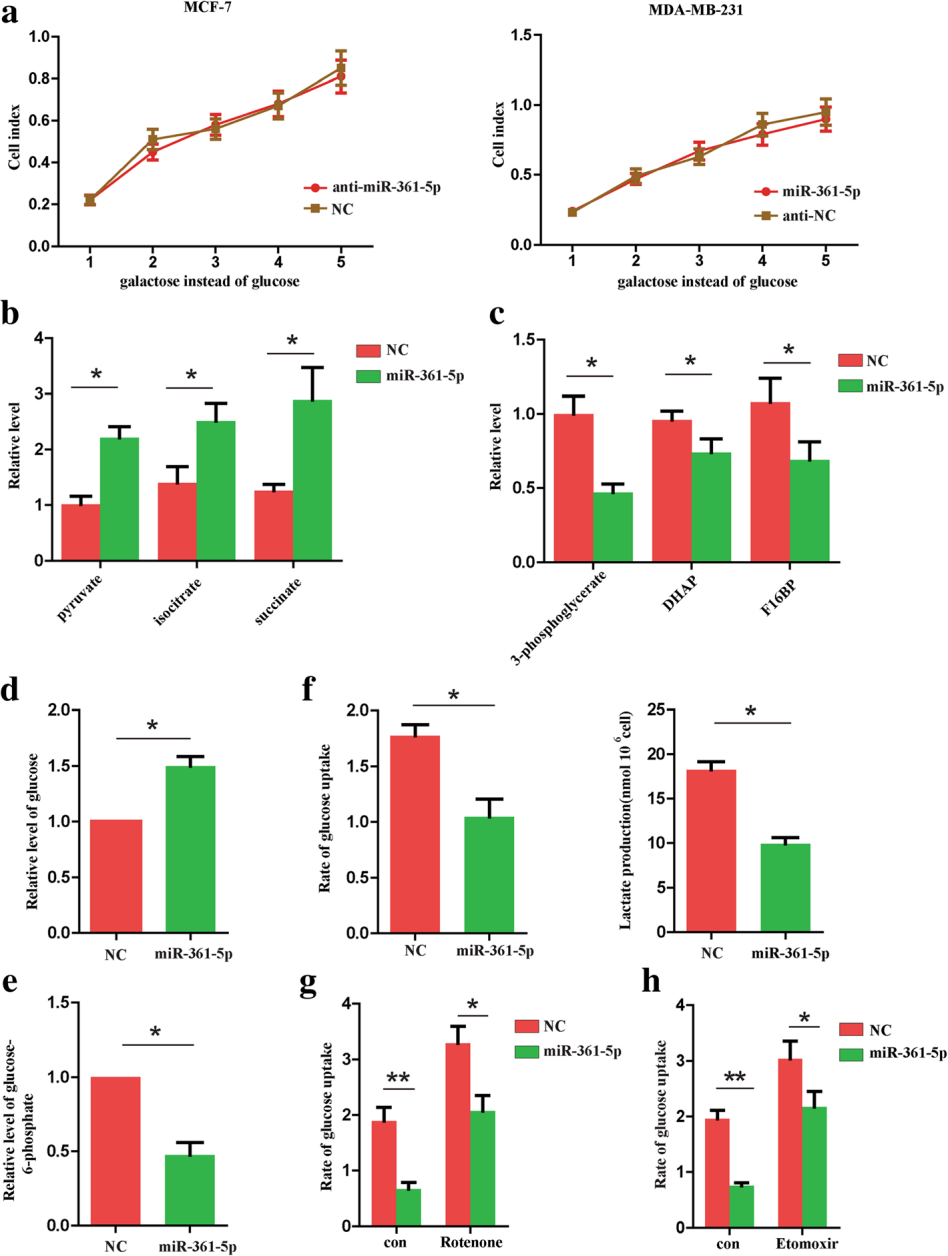
MiR-361-5p inhibits glycolysis and increases mitochondrial metabolism in BC cells. **a** Growth curves of indicated cells cultured in medium containing galactose instead of glucose. Levels of the intermediates in TCA cycle (**b**) and glycolytic metabolism (**c**) in indicated BC cells. Levels of glucose (**d**) and glucose-6-phosphate (**e**) in indicated BC cells. **f** Detection of glucose uptake and lactate production in indicated BC cells. **g** Detection of glucose uptake in indicated BC cells treated with or without rotenone (100 nM). **h** Detection of glucose uptake in indicated BC cells treated with or without etomoxir (50 μg/ml). *P < 0.05, **P < 0.01

To text whether aerobic glycolysis was involved in the process of breast cancer progression, we cultured BC cells using galactose instead of glucose, which could inhibit glycolytic flux and force the cells to rely on mitochondrial oxidative phosphorylation [13]. It was observed that BC cells transfected with anti-NC and anti-miR-361-5p showed similar rate of proliferation under, indicating that glycolysis might be required by the increased BC proliferation. Consistent with these results, there were no difference at proliferation rate between BC cells transfected with NC and miR-361-5p under glucose deprivation (Fig. 3a). However, numbers of invading cells of the anti-miR-361-5p group were higher than that of the NC group, suggesting that glycolysis was not necessary for the enhanced BC invasion (data not shown). Thus, it is possible that miR-361-5p suppressed BC cells growth by regulating the balance between glycolytic metabolism and mitochondrial oxidative phosphorylation. With this idea, we measured the metabolites of miR-361-5p transfected MDA-MB-231 cells. We found that metabolites of the TCA cycle were increased and the intermediates of glycolysis were decreased (Fig. 3b and c). Morever, levels of glucose from miR-361-5p cells were significantly higher compared with control group, whereas levels of the product of glycogenolysis, glucose-6-phosphate, were decreased (Fig. 3d and e). Meanwhile, we found that miR-361-5p cells consumed less glucose and released less lactate into the substrate (Fig. 3f). To conclude, these results suggest that miR-361-5p controls the redirection of BC cellular glucose metabolism and inhibits glycolysis.

To explore whether miR-361-5p suppressed glycolysis due to the increased oxidative capacity, we measured the rate of glucose uptake of miR-361-5p BC cells treated with rotenone (an inhibitor of mitochondrial respiration) or etomoxir (an inhibitor of fatty acid oxidation). We observed that glucose uptake was declined in the presence of rotenone and etomoxir (Fig. 3g and h). These data demonstrated that declined glycolysis in miR-361-5p cells does not attribute solely to increased mitochondrial oxidative phosphorylation.

### FGFR1 mediated the anti-glycolytic function of miR-361-5p by regulating the activity of PDHK1 and LDHA

**Fig. 4:**
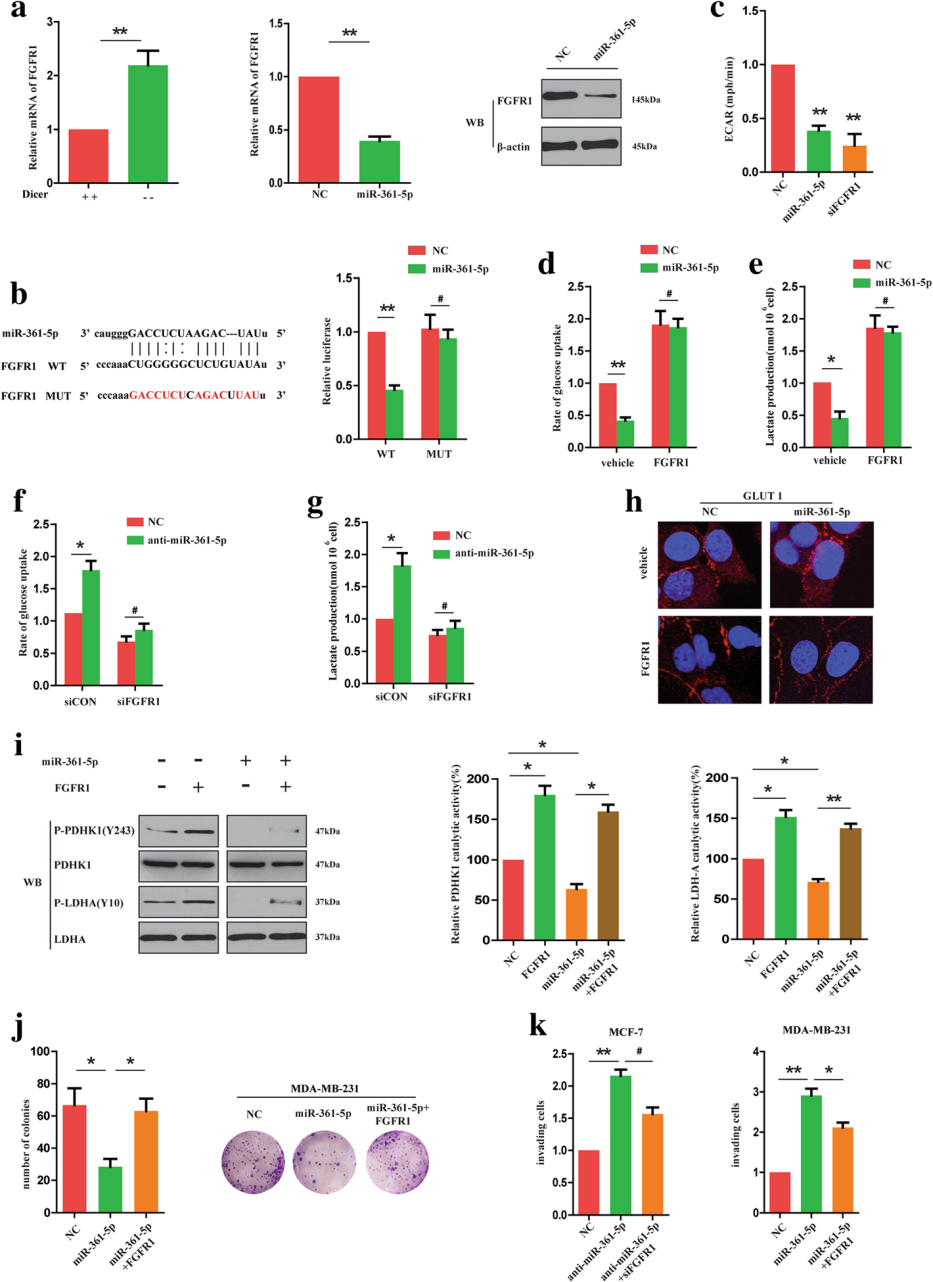
FGFR1 mediated the anti-glycolytic function of miR-361-5p by regulating the activity of PDHK1 and LDHA. **a** Measurement of FGFR1 mRNA expression in Dicer −/− and +/+ (left panel) or WT (middle panel) MEFs by using qRT-PCR. Detection of FGFR1 protein expression in indicated cells (right panel). **b** Luciferase reporter assay for FGFR1 WT and Mut 3’UTRs. **c** ECAR assays for indicated cells. Glucose uptake (**d**) and lactate production (**e**) were detected in BC cells transfected with or without miR-361-5p or FGFR1. Glucose uptake (**f**) and lactate production (**g**) were detected in BC cell transfected with or without anti-miR-361-5p or siFGFR1. **h** Immunofluorescence staining of Glut1 in indicated cells. **i** Western blot analysis was used to detect the phosphorylation and activity of the glycolysis-associated enzymes in MDA-MB-231 cells. **j** Colony formation assays were used to detect the proliferation of indicated cells. **k** Transwell assays were used to detect the effect of FGFR1 on invasion ability of BC cells. *P < 0.05, **P < 0.01

MiRNAs were well known to play crucial roles in various cancers by inhibiting their target genes. Using bioinformatic methods, we happened to find that FGFR1 might be a potential target of miR-361-5p among the numerous candidates, for it was reported to promote aerobic glycolysis by phosphorylating several glycolytic enzymes [14]. Our results showed that Dicer knockdown markedly increased the expression level of FGFR1 mRNA in MEFs, indicating that FGFR1 might be targeted by miRNAs (Fig. 4a). Consistent with these findings, miR-361-5p significantly decreased the expression levels of both FGFR1 mRNA and protein (Fig. 4a). To decide whether FGFR1 was the direct target of miR-361-5p, we constructed luciferase reporters containing FGFR1–3’UTR with a conserved miR-361-5p binding sequence or mutated miR-361-5p binding sequence. It was observed that miR-361-5p inhibited the luciferase activity of WT 3’UTR-reporter, but not the MUT 3’UTR-reporter (Fig. 4b). These results demonstrated that FGFR1 was a direct target of miR-361-5p. To test whether FGFR1 was the functional target of miR-361-5p in inhibiting glycolysis, we measured the extracellular acidification rate (ECAR) which was the surrogate of glycolysis. We found that MDA-MB-231 cells transfected with miR-361-5p or siFGFR1 showed decreased level of extracellular acidification rate compared with control cells (Fig. 4c). Meanwhile, overexpression of FGFR1 in BC cells reverted decreased glucose uptake and lactate production of miR-361-5p-transfected cells (Fig. 4d and e). Conversely, siFGFR1 markedly reverted the enhanced rate of glucose uptake and lactate production in anti-miR-361-5p-transfected BC cells (Fig. 4f and g).

It was reported that GLUT1 could translocate from intracellular membrane compartments to the plasma membrane in epithelial cells to promote glucose uptake [15]. Thus, we used immunofluorescence staining to evaluate the localization of GLUT1 protein. We found that in contrast to control MDA-MB-231 cells, where GULT1 was mostly observed in plasma membrane, GLUT1 was found in the cytoplasm of miR-361-5p-transfected cells (Fig. 4h). However, FGFR1 reversed the effect of miR-361-5p on subcellular translocation of GLUT1 (Fig. 4h). Next, we measured the activation of proteins correlated with glycolytic pathway in MDA-MB-231 cells. Using western blot analysis, we found that miR-361-5p transfection downregulated the phosphorylation of PDHK1 and LDHA in BC cells. However, overexpression of FGFR1 significantly increased the phosphorylation of these proteins in miR-361-5p-overexpressed or control cells (Fig. 4i). Studies have shown that phosphorylation of PDHK1 and LDHA is associated with enhanced enzyme activity and leads to glycolysis increase [16, 17]. Therefore, the results indicated that FGFR1 reverted the anti-glycolytic function of miR-361-5p by upregulating the activity of PDHK1 and LDHA (Fig. 4i). Consistently, colony formation assays indicated that FGFR1 restored the proliferation of miR-361-5p-transfected cells (Fig. 4j). However, siFGFR1 transfection failed to convert the increased invasion ability of anti-miR-361-5p-transfected cells (Fig. 4k), indicating that FGFR1 could not affected the anti-metastatic function of miR-361-5p in BC cells. So we argued that there might be other targets which mediated the anti-metastatic phenotype of miR-361-5p in BC cells.

### MiR-361-5p suppresses the invasion and metastasis of BC by targeting MMP-1

**Fig. 5:**
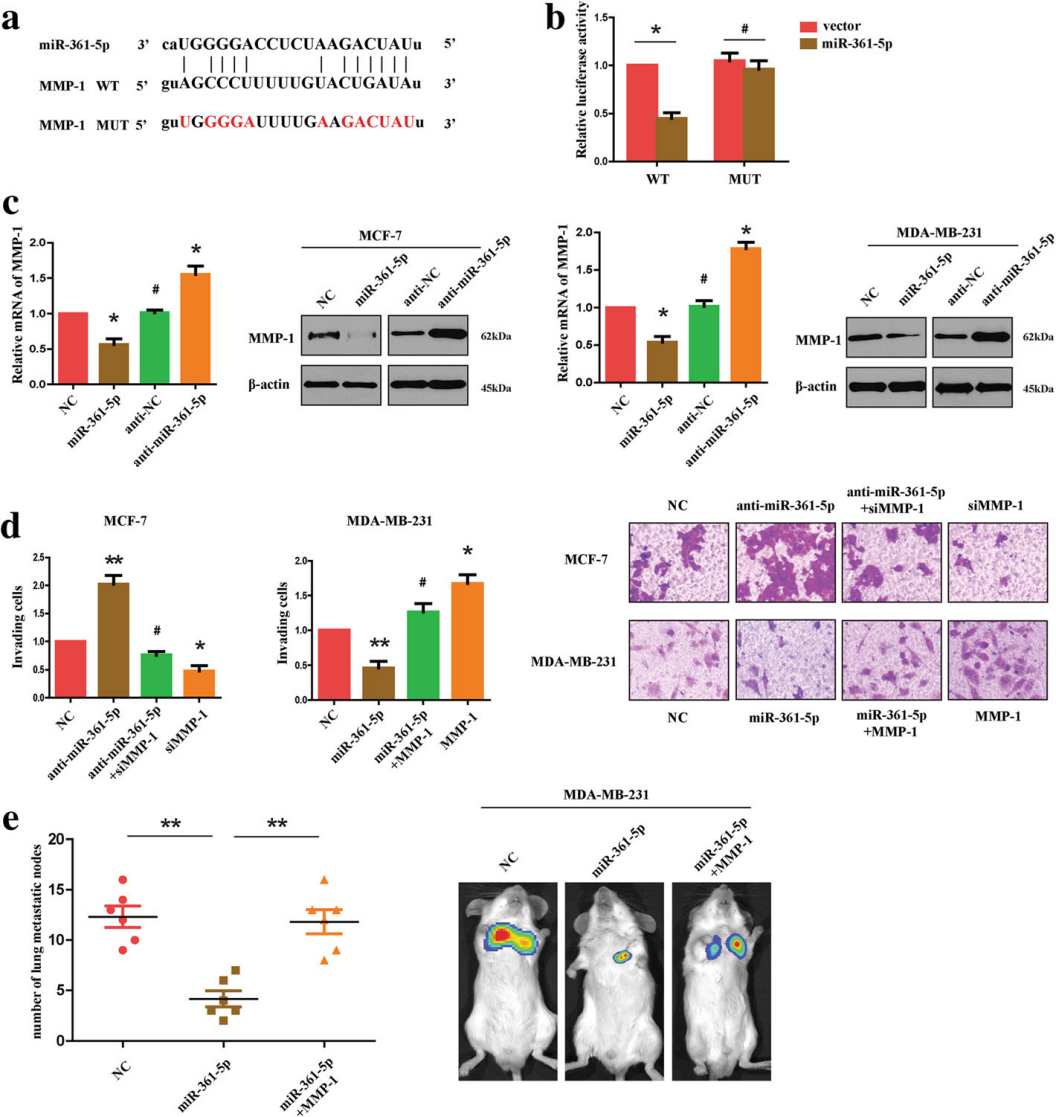
MiR-361-5p suppresses the invasion and metastasis of BC by targeting MMP-1. **a** Schematic diagram of the luciferase reporter containing the WT or MUT miR-361-5p binding sequence in the 3’UTR of MMP-1 mRNA. **b** Luciferase reporter assays for MMP-1 WT and MUT 3’UTR. **c** The expression level of mRNA and protein in BC cells transfected with miR-361-5p or anti-miR-361-5p. **d** Transwell assays were performed to evaluate the effect of MMP-1 on invasion ability of BC cells transfected with miR-361-5p or anit-miR-361-5p. **e** Lung metastasis model was used to detect the metastatic ability of BC cells in SCID mice

To further elucidate the anti-metastatic function of miR-361-5p in BC, bioinformatic methods were used to predict other targets of miR-361-5p. MMP-1 was noted because it contains a putative target sequence of miR-361-5p in the 3’UTR (Fig. 5a) and it was closely correlated with tumor cell invasion [18]. To examine whether MMP-1 was a direct target of miR-361-5p, luciferase reporters containing MMP-1-3’UTR with a conserved miR-361-5p binding sequence or mutated miR-361-5p binding sequence were constructed. We found that miR-361-5p was able to decrease the luciferase activity of WT 3’UTR-reporter, but not the MUT 3’UTR-reporter (Fig. 5b). Consistently, we detected that overexpression of miR-361-5p in BC cells decreased the expression level of MMP-1 mRNA and protein, whereas anti-miR-361-5p increased the expression level of MMP-1 mRNA and protein (Fig. 5c). These results suggested that miR-361-5p directly inhibited MMP-1 expression by binding to its 3′-UTR. Next, we performed restoration assays in BC cells to decide if MMP-1 mediated the anti-metastatic function of miR-361-5p. As shown in Fig. 5d, on one hand, MCF-7 cells transfected with siMMP-1 showed decreased invasion ability compared with control cells, on the other hand, siMMP-1 restored the enhanced invasion ability of miR-361-5p-inhibited MCF-7 cells. In contrast, MMP-1 overexpression in MDA-MB-231 cells raised the number of invading cells and restored the decreased the ability of invasion in miR-361-5p-transfected cells. Similarly, the results of in vivo assays showed that MMP-1 reverted the anti-metastasis phenotype of miR-361-5p in BC cells (Fig. 5e). Altogether, these results indicated that miR-361-5p suppressed the invasion and metastasis of BC by targeting MMP-1.

### Clinical relevance of miR-361-5p, FGFR1 and MMP-1 expression in BC patients

**Fig. 6:**
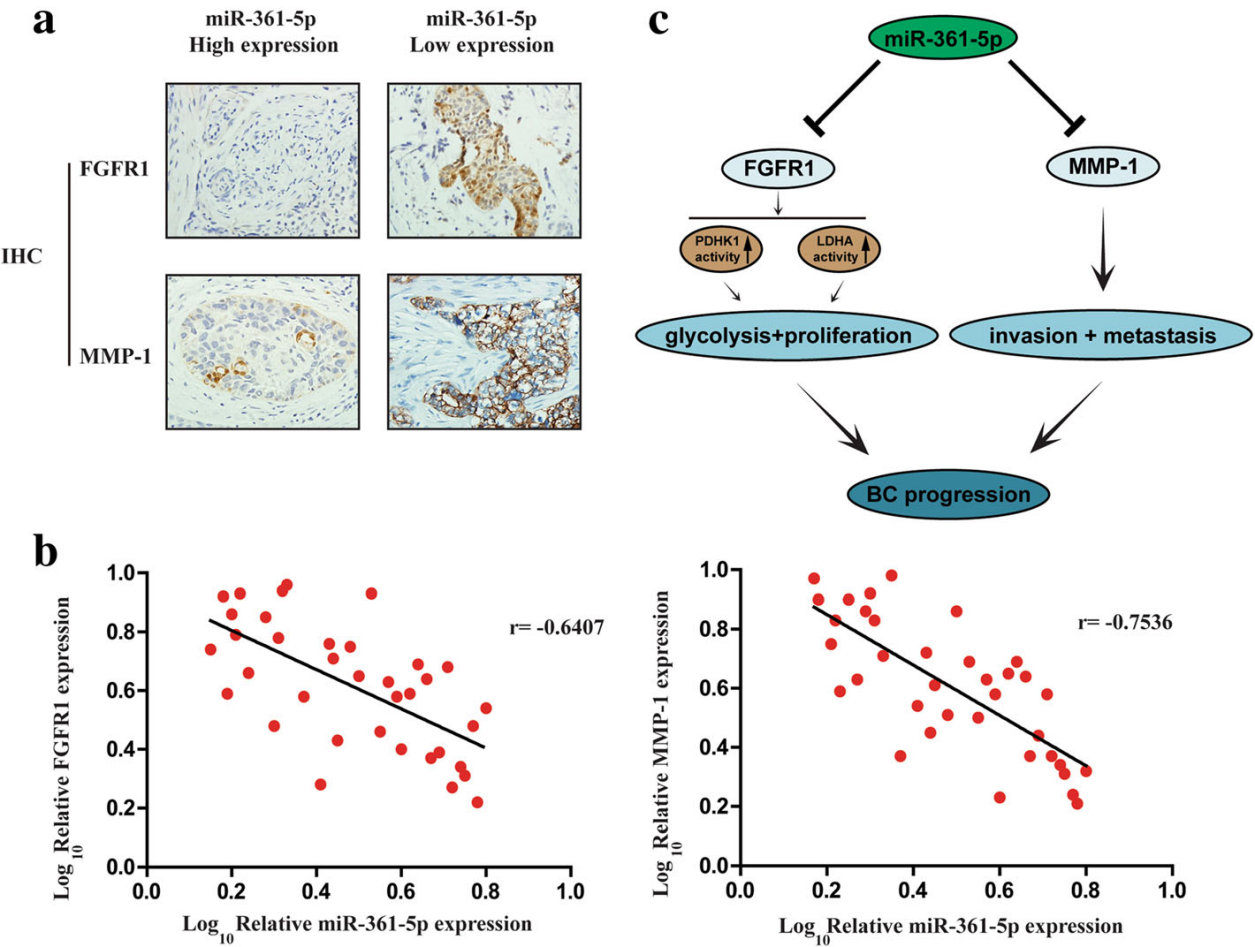
Clinical relevance of miR-361-5p, FGFR1 and MMP-1 expression in BC patients. **a** Representative IHC staining images of FGFR1 and MMP-1 expression in high/low miR-361-5p expression BC tissues. **b** The relationship between miR-361-5p expression and FGFR1 (left panel) and MMP-1 (right panel) expression in clinical samples. **c** Schematic diagram of the mechanism that miR-361-5p inhibits BC progression

Given that miR-361-5p downregulated FGFR1 and MMP-1 in BC cells to suppress tumor progression, we next explored whether this relationship existed in clinical samples. We found that BC tissues with high miR-361-5p expression showed low IHC score of FGFR1 and MMP-1. Reversely, BC tissues with low miR-361-5p expression tended to show high expression of FGFR1 and MMP-1 (Fig. 6a). A reverse relationship of expression between miR-361-5p and FGFR1 or MMP-1 was observed (Fig. 6b). These data support the mechanism that miR-361-5p targets FGFR1 and MMP-1 respectively, inhibits BC cell glycolysis and proliferation, invasion and metastasis, and finally suppresses BC progression (Fig. 6c).

## Discussion

In this study, we found that the expression of miR-361-5p was downregulated in breast cancer and was associated with poor prognosis. As a tumor suppressor, miR-361-5p inhibited BC cells aerobic glycolysis and proliferation by directly targeting FGFR1, a promoter of glycolytic pathway. Meanwhile, miR-361-5p also targeted MMP-1 to suppress BC cells invasion and metastasis. Thus, our results provide evidence that miR-361-5p inhibits glycolytic metabolism, proliferation and invasion of BC cells and reveal the specific regulatory mechanism of miR-361-5p in BC, suggesting that miR-361-5p and its target genes may serve as potential therapeutic targets for BC patients.

Emerging evidence has demonstrated that microRNAs play crucial roles in multiple biological and pathological processes of cancer, including tumor cell proliferation, invasion and metastasis [19]. It has been reported that some miRNAs acted as tumor regulators and could reduce the expression of many target oncogenes [20]. MiR-361-5p, known as a tumor suppressor, was reported to play functional roles in several cancers [21, 22]. However, the role of miR-361-5p and its regulatory mechanism in BC have rarely been discussed. By coincidence, our previous study has demonstrated that miR-361-5p inhibits the malignant phenotype of colorectal and gastric cancer by targeting SND1 [12]. Morever, another recent study also showed that increased expression of miR-361-5p predicted improved BC survival [23]. Similarly, Cao et al. reported that the clinical outcomes of patients with positive miR-361-5p expression was better than that of patients with negative miR-361-5p expression [11]. Consistent with those studies, we observed that miR-361-5p was down-regulated in breast cancer compared with normal breast tissue, and decreased miR-361-5p expression was correlated with poor DFS. Nevertheless, we found that decreased miR-361-5p expression was also correlated with larger tumor size, lymph node metastasis and higher TNM stage, which was not significantly evidenced in the previous study. This difference in results might be caused by the distinct grouping modes between the two studies. In addition, we were the first to analyze the functional roles of miR-361-5p in BC cells and found that miR-361-5p suppressed breast cancer cells proliferation, invasion and metastasis both in vitro and in vivo. Based on these results, miR-361-5p could be recognized as a tumor suppressive miRNA in BC.

The type of glucose metabolism in tumors can shift widely between glycolysis and OXPHOS [24]. Considering the fact that increased glucose uptake is associated with enhanced biosynthetic metabolism, the specific molecular mechanisms that upregulate glycolysis and anabolic biosynthesis are of great importance. Accumulating evidence showed that dysregulation of multiple metabolic enzymes might contribute to the aerobic glycolysis process, including glucose transporter 1 (GLUT1), pyruvate dehydrogenase kinase 1 (PDHK1) and lactate dehydrogenase (LDHA) [25–27]. Previous studies found that phosphorylation of LDHA and PDHK1 by FGFR1 was common in diverse cancers which could activate LDHA and PDHK1 and promote glycolysis [16, 17]. Consistent with these studies, we found that FGFR1 reverted the anti-glycolytic function of miR-361-5p by upregulating the activity of PDHK1 and LDHA, which enrich the mechanism that miR-361-5p inhibited BC cells glycolysis and proliferation. However, there might be many other questions remain to be solved, such as possible involvement of other targets and the detailed post-translational modifications of the metabolic enzymes. The investigation of these issues will no doubt enrich the mechanism that miR-361-5p and FGFR1 regulate BC cells glycolysis and proliferation.

Due to the fact that FGFR1 overexpression only partly converted the invasion ability of miR-361-5p-transfected cells, we questioned the possibility that another target of miR-361-5p might mediate its anti-metastatic phenotype. MMP-1, which belongs to a large family of peptidases, was identified to cleave the components of extracelluar matrix and play crucial roles in the movement of epithelial cells [28]. Consistent with that, we demonstrated that MMP-1 was a direct functional target of miR-361-5p and mediated the anti-metastatic phenotype of miR-361-5p in BC cells. Interestingly, a previous study showed that the activation of FGFR1 might be induced in response to MMP-1 [29]. Thus, the regulatory relationship between FGFR1 and MMP-1 downstream to miR-361-5p in breast cancer remains to be investigated in the future. Meanwhile, considering that several studies reported miR-361-5p to act its function by targeting some other factors, such as Twist1, VEGFA and FOXM1 [30–32], it remains to be explored whether other targets may contribute to the anti-tumor effect of miR-361-5p.

## Conclusion

Our study found that miR-361-5p was down-regulated in breast cancer tissues and the expression of miR-361-5p was positively associated with prognosis in BC patients. Functional studies showed that overexpression of miR-361-5p suppressed the glycolysis and proliferation of breast cancer cells by targeting FGFR1, the invasion and metastasis by targeting MMP-1. An inverse expression pattern was also found between miR-361-5p and FGFR1 or MMP-1 in clinical samples. Our results suggest that miR-361-5p and its target genes may serve as therapeutic targets in breast cancer treatment.

## Abbreviations

BC: Breast cancer
DFS: Disease-free survival
EACR: Extracellular acidification rate
EMT: Epithelial-mesenchymal transition
FGFR1: Fibroblast growth factor receptor 1
GLUT1: Glucose transporter 1
LDHA: Lactate dehydrogenase A
microRNA: miRNA
MMP-1: Matrix metalloproteinase-1
ORF: Opening reading frame
OXPHOS: Oxidative phosphorylation
PDHK1: Pyruvate dehydrogenase kinase 1
PTEN: Phosphatase and tension homolog deleted on chromosome ten
SND1: Staphylococcal nuclease domain-containing protein 1
UTR: Untranslated region
